# An Overview of Recent Application of Medical Infrared Thermography in Sports Medicine in Austria

**DOI:** 10.3390/s100504700

**Published:** 2010-05-07

**Authors:** Carolin Hildebrandt, Christian Raschner, Kurt Ammer

**Affiliations:** 1 Department of Sport Science, University of Innsbruck, Fuerstenweg 185, A-6020 Innsbruck, Austria; E-Mail: christian.raschner@uibk.ac.at; 2 Institute for Physical Medicine and Rehabilitation of the Hanuschspital, Heinrich-Collin-Street 30, A-1140 Vienna, Austria; E-Mail: kammer1950@aol.com

**Keywords:** thermal imaging, injury management, knee, infrared sensor technology

## Abstract

Medical infrared thermography (MIT) is used for analyzing physiological functions related to skin temperature. Technological advances have made MIT a reliable medical measurement tool. This paper provides an overview of MIT’s technical requirements and usefulness in sports medicine, with a special focus on overuse and traumatic knee injuries. Case studies are used to illustrate the clinical applicability and limitations of MIT. It is concluded that MIT is a non-invasive, non-radiating, low cost detection tool which should be applied for pre-scanning athletes in sports medicine.

## 1. Introduction

Medical infrared thermography (MIT) provides a non-invasive and non-radiating analysis tool for analyzing physiological functions related to the control of skin-temperature. This rapidly developing technology is used to detect and locate thermal abnormalities characterized by an increase or decrease found at the skin surface. The technique involves the detection of infrared radiation that can be directly correlated with the temperature distribution of a defined body region [1].

An injury is often related with variations in blood flow and these in turn can affect the skin temperature. Inflammation leads to hyperthermia, whereas degeneration, reduced muscular activity and poor perfusion may cause a hypothermic pattern [2]. There are several applications of MIT in the field of human medicine, such as neurological disorders [3], open- heart surgery [4], vascular diseases [5], reflex sympathetic dystrophy syndrome [6], urology problems [7] and mass fever screening [8]. Much research has been focused on the successful evaluation of breast cancer [9]. According to Ng [10] breast thermography has achieved an average sensitivity and specificity of 90%. He reported that an abnormal breast thermogram is a significant biological marker for breast cancer. One possible explanation is that increased blood flow due to the vascular proliferation that results from angiogenesis is associated with tumors [11]. Reduced skin temperature has also been implicated in musculoskeletal disorder (MSD). In fact, a cold skin pattern around ankle sprains indicates a poor prognosis and a long recovery time [12].

Infrared sensor technology also contributes to the field of injury management in athletic animals [13–16]. Anatomical and physiological similarities between animals and humans may imply that modern infrared sensor technology can provide significant information for the functional management of injuries in human athletes. However, there is scant scientific evidence of its successful application in the field of human sports medicine.

High performance training pushes the locomotor system to the edge of its anatomical and physiological limits. The knee is a weak link and is the most frequently affected joint in sports. Knee injuries are common in skiing and sports that involve jumping and abrupt direction changes [17]. Current trends indicate that in Austria, one of the top ski countries, the number of participants in competitive alpine skiing is greatly increasing, triggering a proliferation of knee injuries [18]. The incidence of long-term effects, such as osteoarthritis, are alarming.

These injuries usually involve a long, costly rehabilitation period and are often career-ending for athletes. The need for further research in the field of injury prevention and management is crucial to counteract severe skiing injuries.

## 2. International Status of Medical Infrared Imaging

MIT has been recognized by the American Medical Association council as a feasible diagnostic tool since 1987 and was recently acknowledged by the American Academy of Medical Infrared Imaging. Various groups and associations promote the proper application of thermal imaging in the practice of sports medicine. These groups include the European Association of Thermology, the United Kingdom Thermography Association, and the Northern Norwegian Centre for Medical Thermography, the American Academy of Thermology and the German Society of Thermography and Regulation Medicine (DGTR) as one of the oldest medical thermography society. The overall aim of these groups is to further improve reliable standardized methods and to develop appropriate protocols for clinical application.

The usefulness of MIT in sports medicine has been noted often [19]. However, some doubts about the technology highlight the necessity of doing further research. The major argument is whether MIT can accurately determine thermal variations to enable sufficient quantitative analyses [20]. Proponents of MIT state that “state-of-the-art” computerized systems using complex statistical data analysis ensure high quality results [21] and that thermal sensitivity has increased, creating a new dimension that should be exploited and applied [22].

The absence of a standardized reference images is also a problem [23]. A research group from the University of Glamorgan is currently conducting research to determine “normal” thermograms by creating an “*Infrared Atlas of Normal Human Skin Temperature Distribution”.* Well-designed research studies can address these issues and help to resolve them.

## 3. Principles and Technique of Infrared Thermography

### 3.1. Electromagnetic Spectrum

There are several medical imaging modalities within the electromagnetic spectrum, which is defined as the range of electromagnetic radiation frequencies. Depending on their physical principles, these various techniques mainly provide anatomical information.

MIT is essentially a digital two-dimensional imaging technique that provides data about the physiology of tissues [24]. Unlike most diagnostic modalities, MIT is non-invasive. The question is whether physiological images can change prior to anatomic disruption. Specific software makes it possible to incorporate anatomical and physiological information by image fusion, which helps to localize the affected area and extent of the injury.

All images are obtained through the energy from the human tissue, leading to a classification based on the energy applied to the body. The energy content of the emission is related to the wavelength of the radiation.

Regarding the spectral region, human skin is a black body radiator with an emissivity factor of 0.98 [25] and is therefore a perfect emitter of infrared radiation at room temperature. Planck’s law describes the characteristics of infrared radiation emitted by an object in terms of spectral radiant emittance [26].

**Formula 1.** Planck’s radiation law. $W(\lambda ,T)=\bar{\frac{2{\pi \mathrm{hc}}^{2}}{\lambda^{4}}}{\left[\text{exp}\left(\frac{\mathrm{hc}}{\lambda \mathrm{kT}}\right)-1\right]}^{-1}{\mathrm{Wcm}}^{-2}\mu m^{-1}$

H (Planck’s constant) = 6.6256 × 10^−34^ Js
K (Boltzmann’s constant) = 1.38054 × 10^−23^ WsK^−1^
C (velocity of light in vacuum) = 2.9979 × 10^8^ ms^−1^
μ = wavelength in μm
T = temperature in K

Human skin emits infrared radiation mainly in the wavelength range of 2–20 μm with an average peak of 9–10 μm [25]. Based on Plank’s Law roughly 90% of the emitted infrared radiation in humans is of longer wavelength (8–15 μm).

**Figure 1.**
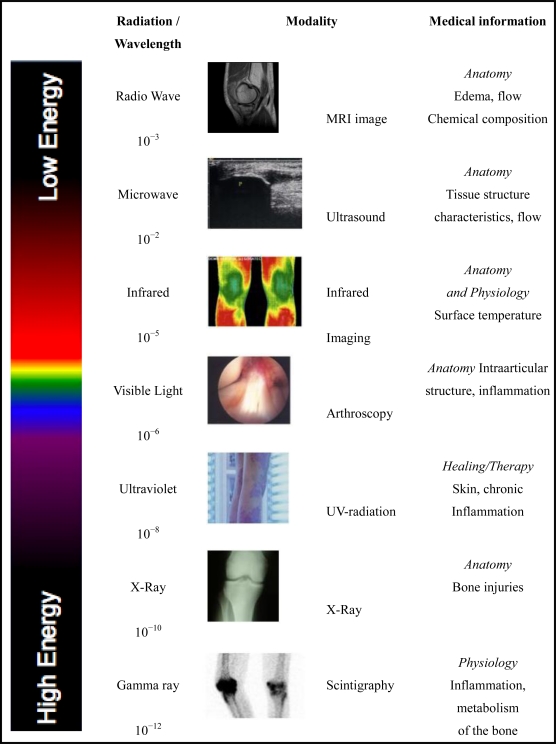
Typical imaging modalities within the electromagnetic spectrum.

Figure 1 gives an overview of the medical imaging modalities used within the electromagnetic spectrum.

## 4. Infrared Radiation

Emissivity refers to an object’s ability to emit radiation [27]. Infrared cameras generate images based on the amount of heat dissipated at the surface by infrared radiation. The technology is a sophisticated way of receiving electromagnetic radiation and converting it into electrical signals. These signals are finally displayed in gray shades or colors which represents temperature values. Human heat energy is transferred to the environment via four mechanisms [28]:

1. Conduction: the transfer of heat energy via tissue layer by contact between two bodies of different temperatures;
2. Convection: the heat change between the skin and the surroundings; and
3. Radiation: a transfer of heat that does not require a medium. The energy is transferred between two separate objects at different temperatures via electromagnetic waves (photons)
4. Sweat Evaporation: which is the main mechanism for heat dissipation during exercise? The conversion of liquid into vapor allows the body to regulate its temperature. Evaporation results in a decrease of surface temperature.

The constructed thermogram yields a quantitative and qualitative temperature map of the surface temperature, which can be related to distinct pathological condition and blood flow.

Different to a single detector thermal camera, focal plane array detectors generate thermal images of high resolution without a mechanical scan mechanism. These cameras operate in the long wave infrared region (8–15 μm) with the advantage that they are less affected by sunlight compared to the shorter waves.

### 4.1. The 21st Century Technique

The medical usefulness of infrared thermography has been proven over the last several years but has largely been done without the advantage of 21st century techniques [29].

A new generation of high-resolution cameras has been developed, leading to improved diagnostic capability. Changes in the thermal pattern that may be very small but still meaningful can be properly assessed.

These technical enhancements have made infrared thermography into a reliable and powerful measurement tool [30]. It has opened opportunities for very precise measurements by imaging very subtle changes in skin surface temperature. Table 1 gives an overview of recent technical developments.

### 4.2. Recommended Requirements for Human Medicine

The thermal imaging group from the University of Glamorgan has recently published a battery of tests for checking the reliability of an infrared camera [23,31].

**Figure 2.**
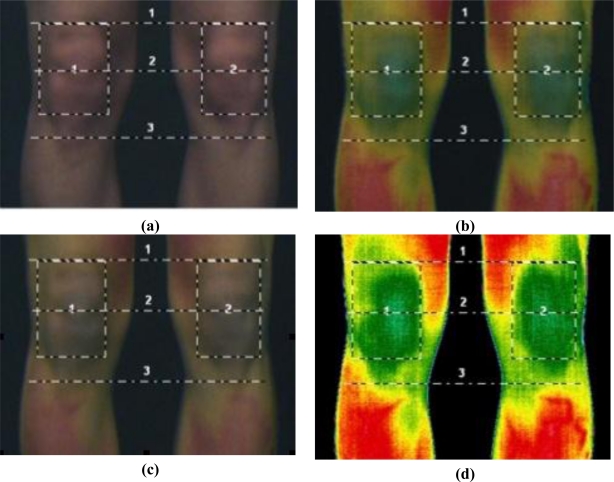
Process of image fusion. (a) Anatomical image; (b) Image fusion first step; (c) Image fusion second step; (d) Infrared Image.

An infrared camera suitable for evaluating human skin profiles should have the following [31–33]:

1. High Spatial resolution which reflects the separation between two nearby spots. A resolution of 320 (horizontal) × 240 (vertical) pixel is the minimum requirement. The spatial resolution is very dependent on image focusing.
2. High Thermal resolution as an expression of sensitivity, defined as the minimum temperature difference that can be measured at two distinct spots.
3. Medical CE certification is recommended: As soon as a temperature value in degree celcius is stated, the device is classified as a medical modality with a measuring function and should be signed by a specific CE approval.
4. Narrow Calibration range accustomed to the human temperature range (*i.e.*, 20–40 °C) assures more detailed temperature readings.
5. Medical examination software including an export function, for medical analysis report and well-designed software tools for data analysis and image fusion (Figure 2).

In general, an area read-out of at least 8 × 8 pixels should be used instead of hot spot measurements [34]. According to Mayr [35] a line shaped and rectangular form (as seen above) is possible when assessing side-to-side differences. The upper and lower edge of the patellae as well as the tibial tuberosity can be clearly defined by image fusion and is therefore recommended for use as an anatomic marker system [36].

## 5. Reliability Study

Reliable measurements have a substantial impact on the diagnosis and interpretation of pathophysiological abnormalities. Many investigations about reliability have focused on equipment and errors related to the physical principles of the technique [31,37]. In addition to technical variations, biological changes such as the circadian rhythm may also contribute noise to the measurements [38].

The reproducibility of the thermal pattern is important if MIT is to be used as a screening tool for injuries. Selfe *et al*. [36] conducted a study of inter-rater reliability and determined that MIT generated adequately reliable thermal patterns from the anterior knee.

**Figure 3.**
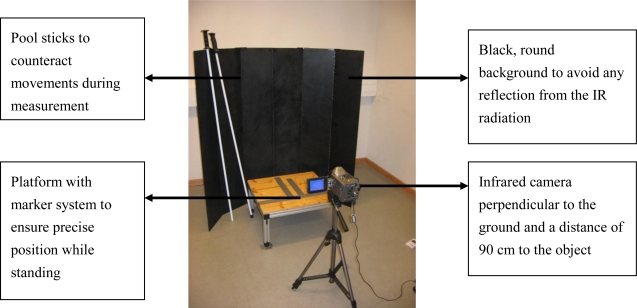
Set up for measurement.

The amount of heat emitted from the knee is a complex phenomenon that is influenced by many factors and comparing images over time requires good standardization methods and quality assurance [39]. We conducted a preliminary study to evaluate the day-to-day repeatability [40]. To guarantee reliable measurements, a standardized setup was used as shown in Figure 3.

### 5.1. Methods of Reliability Study

Mean temperature readings of the anterior aspect of the knee of 15 subjects were analyzed. To eliminate inter-rater error, the same person carried out the measurements each time. The examination was conducted according to the “Glamorgan Protocol” which was established to ensure quality control when using MIT for medical applications [23].

To provide consistency for repeated measurements, anatomical landmarks were marked on the subject to delineate the region of interest for data capture.

### 5.2. Results

While high individual variations in knee temperature between subjects were noted, low variations between day-to-day measurements indicated the overall stable temperature of the knee. The one-way random intra-class correlation coefficient (ICC) indicated good intra-examiner reliability for absolute values of mean temperature for the right leg and moderately good reproducibility for the left leg (Table 2).

In agreement with other studies, we concluded that MIT is a promising evaluation tool when administered under standardized conditions [1,39–41]. The results of these studies were recently published in the journal *Thermology International* and provide a more detailed description of methods [40].

## 6. Clinical Application in Alpine Skiing

Previous research has demonstrated that thermal images from the two sides of the body are usually symmetrical [42,43]. Any significant asymmetry of more than 0.7 °C can be defined as abnormal and may indicate a physiologic or anatomical variant in the loco-motor system. By comparing one side with the other, it may be possible to detect sub clinical problems before they are clinically relevant.

One of the most beneficial contributions of MIT to sports medicine may be in the field of preventive medicine. Turner *et al*. [14] examined tendonitis in racehorses and thermographically detected hot spots two weeks before clinical evidence of swelling, pain and lameness. Early detection of abnormal changes in the tissues is important to counteract overuse injuries. The knee is exposed to a lot of physical stress during the alpine skiing competition season. The so-called “little traumatologies” are very frequent; therefore, their early detection is important [44]. However, it must be emphasized that the primary goal is to detect irregularities in the symmetry of temperature distribution rather than the measurement and comparison of absolute temperatures.

There are currently no quick screening tools that are sufficiently predictive of impending symptoms. To verify the thesis that MIT could predict symptoms, we conducted a pre-season measurement of 35 female and 52 male junior alpine ski racers. This study included likewise athletes who were in rehabilitation after traumatic and acute injuries.

### 6.1. Methods

Following an acclimatization period of 20 minutes, we recorded an image of the anterior/posterior and medial/lateral aspect of both knees with an infrared camera (TVS500EX). A fixed distance of 95 cm from the camera to the subject was used.

Data were stored and analyzed with the iREPORT 2007 software, provided by the GORATEC GmbH. All images were corrected using an emissivity factor of 0.98. Image fusion was used to identify the area of interest. The room temperature remained constant ranging from 21.5–22.3 °C. Equally the relative humidity showed stable values over time (35–38%).

Infrared images were taken twice to get pre- and postseason measurements. Thermographic evaluation was done according to the guidelines prepared by the medical members of the American Academy of Thermology (AAT) and the Glamorgan protocol [23], which incorporates the following seven aspects:

- Patient communication
- Patient preparation
- Patient assessment
- Examination guidelines
- Review of the imaging examination
- Presentation of the findings
- Exam time recommendation continuing professional education

An experienced team of sports physiotherapists conducted the musculoskeletal examination to obtain data about the functional aspects of the knees. Each subject had to fill in a questionnaire to get additional information about:

- Name, age, sex
- Sport history including information about training performed in the previous 7 days
- Health status
- Nutritional status
- Menstrual cycle

### 6.2. Case Studies

#### 6.2.1. Overuse Injuries

**Figure 4.**
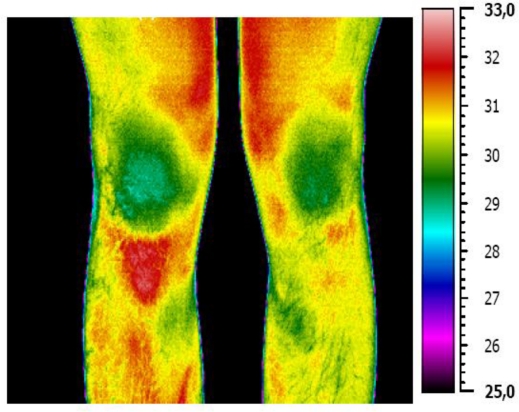
Infrared image of the anterior aspect of the knees (Enthesopathy of the ligamentum patellae affects the right knee). The temperature scale applies for each infrared image below.

A common problem in alpine skiing is the occurrence of overuse injuries such as patellae tendinopathy, which is characterized by swelling, pain and tenderness above the tibial tuberosity [45]. This regional problem becomes apparent in the form of a hyperthermic pattern, as can be seen in Figure 4 in which the right knee is affected. The preseason training program includes excessive jumps, leading to mechanical strain and overuse of the patella tendon.

In this study, a total of seven athletes showed symptoms of regional overuse reactions. The symptomatic athletes had a mean side temperature differences of 1.4 °C (±0.58 °C). The normal temperature range of the eight non-injured athletes showed a side-to-side variation of 0.3 °C (±0.61 °C).

Four of the injured athletes reported pain, while the others were asymptomatic at that time. However, physical examination of the knee revealed that this hyperthermia was associated with a low threshold for pressure pain, as previously described in the literature [46].

Early detection and subsequent early therapy intervention program can reduce the severity of symptoms. Furthermore, the detection of at-risk athletes makes it possible to adjust their training program.

#### 6.2.2. Traumatic Injuries

**Figure 5.**
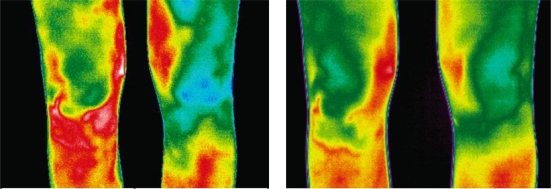
Infrared image of the anterior aspect of the knees (ACL rupture in the right knee).

Epidemiological studies have shown a high incidence of serious knee injuries among alpine skiers, with the most common injury being the rupture of the anterior cruciate ligament (ACL) [47,48]. In Figure 5, the image on the left side was taken 6 weeks follow–ing an isolated ACL rupture of the right knee. The massive hyperthermia around the lower patellae represents the inflammation process, which is accompanied by swelling and pain. The image on the right side shows the same knee following 6 months of extensive rehabilitation program.

A clear decline in swelling and inflammation can be seen. However, pain sensation is still present on the medial aspect of the right knee, as indicated by the hyperthermic area.

**Figure 6.**
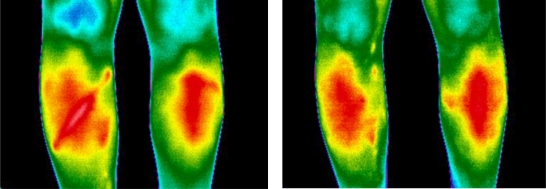
Infrared image of the anterior aspect of the knees (fracture tibia and fibulae in the right knee).

Severe alpine skiing accidents may result in serious injuries such as fractures. In Figure 6, the infrared image on the right side was taken 3 months after a combined fracture of the tibia and fibula with intramedulary nailing. This injury resulted in a clear demarcation and localization making it possible to define the extent of the high metabolic activity in structures involved.

Following treatment, no clear differences of the temperature distribution between the two sides could be noted. In conjunction with the clinical examination, the complete recovery was confirmed. However, a high temperature on the shank can be noted on both legs, possibility due to increased muscular activity. Follow up imaging is required for long-term evaluation.

The incidence of soft tissue injuries such as muscle strains is relatively small in alpine skiing [44]. However, these injuries are a strong risk factor for future strain injury to the same muscle. Full recovery needs to be assured and may be visualized threw thermal imaging.

It is very important to understand the pathophysiology, phases and time frame of normal tissue healing of traumatic injuries. Regular MIT measurements within the rehabilitation process provide information about the ongoing healing process and improve the therapist’s ability to create an adapted rehabilitation and treatment program. Infrared images may give full recovery information by indicating by low side-to-side differences and decreasing the likelihood of re-injury by returning to the sport too quickly.

These results are based on primary investigations and can be regarded as a first step to provide a scientific database for validating overuse and acute knee injuries when examined with MIT. Further research is intended to distinguish between normal and abnormal temperature patterns [49].

## 7. Limitations and Advantages of Infrared Imaging

The efficiency, safety and low cost of MIT make it an auxiliary tool in medical imaging and diagnostics [19,50]. It can be applied without any objections because this non-invasive technique works without damaging radiation. It has the potential for performing *in vivo* diagnosis on tissues without the need of sample excision; hence, it can be regarded as a passive measurement [30]. Furthermore, the resulting real time information can be used as instant feedback for the patient or athlete. Innovative concepts such as dynamic thermal imaging will be applied to further explore skin thermal properties in response to stresses such as excessive jumping performance and training, as one important part of specific training in alpine skiing [51].

Cutaneous temperature changes during exercise can now be detected by functional thermal imaging using state-of -the art infrared sensor arrays and may provide additional useful data [52–55].

However, infrared thermography becomes even more useful when its limitations are known. For future consideration, it is important to know that this can provide physiological information but cannot define aetiologies and local anatomy. The Individual variability combined with the complex character of thermoregulation limits the interpretation. The lack of specificity makes it necessary to combine these measurements with other, more structural modalities (X-ray, computed tomography), rather than using it as a replacement.

The biggest challenge is to combine the anatomical and physiological information given by the thermal pattern of the skin surface. The use of instrumented techniques to measure circulatory conditions must be considered.

Automated overlay of infrared and visual medical images as well as automated target recognition are also being actively studied [56,57]. By applying these new techniques we may reduce operator dependence and enhance accuracy and objectivity.

## 8. Conclusions

Thermal imaging in medicine is not new, but early investigation with old and insufficient techniques has led to work with dubious results. Recent work with modern 21st century technology has demonstrated the value of MIT in medical application when used as an auxiliary tool. Knowledge about thermoregulation, anatomy, physiology, morphology and pathophysiological processes is important to counteract inaccurate diagnoses.

The aim of this technique is not to be a substitute for clinical examination but to enhance it. Further research and follow-up studies are warranted to create databases for clinical measurements and further determine its viability in real-world medical settings. Empirical evidence of correlation between pathology and infrared imaging is essential to further predict the value of MIR. It should be used as a multidisciplinary assessment tool by experts from different fields.

Based on the advantages of MIT as a non-invasive, non-radiating, low cost first-line detection modality, it should be applied in the field of sports medicine as a pre-scan team assessment tool. The extension of sport specific databases may further contribute to the detection of high risk athletes and help them to start early intervention.

## Figures and Tables

**Table 1.** Development of infrared sensor technology.

**OLD TECHNIQUE**	**NEW TECHNIQUE**
Liquid detector cooling	Uncooled camera technology
Single element detector	Focal plane array detector
Slow mechanical scan mechanism	Real time, high-speed imaging with multi elements arrays
Low resolution camera	High-resolution camera
Analogue conversion and computing	Digital conversion and computing, electronic transfer of images from camera to PC in real time
No sufficient knowledge about standardization methods	Standardization protocols and recommendations for medical use
Gray shade images	Color visible images
Expensive, big in size, not mobile	Affordable, smaller and fully mobile
Predominantly low sensitivity	Improved sensitivity (0.02 degrees celcius)
Insufficient software and tools	User-friendly image processing software

**Table 2.** Intra-examiner reproducibility of mean knee temperature

**Intra-examiner reliability of the mean Temperature (n = 15)**		
	ICC	Range^a^
Right leg	0.85	0.61–0.94
Left leg	0.75	0.41–0.90

a 95% confidence intervals.

**Table 3.** Temperature readings (°C) of the area above the tibial tuberosity (n = 7).

	**Affected knee**	**Non-affected knee**	**Temperature differences**
**Mean**	32.8 (± 0.48)	31.1 (± 0.32)	1.4 (± 0.58)
**Minimum**	31.4 (± 0.43)	30.3 (± 0.41)	0.8 (± 0.31)
**Maximum**	33.4 (± 0.39)	32.1 (± 0.60)	1.3 (± 0.64)
